# Clinical Review, and Hospital Reports

**Published:** 1844-07-01

**Authors:** 


### Clinical Review.

---

REPORT OF CASES ILLUSTRATING DISEASES OF THE BRAIN AND NERVOUS SYSTEM. [Guy's Hospital Reports, April 1844.]

BAGLIVI seemed to think that diseases of the lungs offered the greatest difficulties in the way of diagnosis. Thanks to auscultation and percussion, affections of the respiratory apparatus are now more easily distinguished than those of any other internal organ—excepting, perhaps, the heart. Undoubtedly the maladies, functional as well as organic, of the encephalon, are the most puzzling of the complaints which present themselves to the medical practitioner.

The paper on Cerebral Affections, in Guy's Hospital Reports, is without the name of any author or reporter. But it is one of some importance, and demands notice in a practical journal.

*Case* 1. *Arachnitis.*—A female aged 19, a servant, was admitted, after an illness of a fortnight, labouring under pain in the head and loins, fever, and occasional delirium. The usual remedies had been used. There was strabismus—injected tongue, coated yellow—dry skin—pupils rather contracted, and unaffected by light, pulse small and frequent—abdomen rather tender on pressure—answered questions somewhat incoherently.

"She was ordered to have beef-tea and arrow-root; and after taking three grains of calomel, followed by half an ounce of castor-oil, the following treatment was prescribed:—

Hyd. c̄ Cretâ gr. iij. ter die sumend.
Ung. Hydrarg. ℈i. singulis axillis bis die infricand.
Empl. Cantharidis pone singulas aures applic.
Sinapismata singulis pedibus.

The bowels were twice acted on. She passed a very restless night, with much incoherence; the gums were tender on the day following; the pulse 112, very small and weak; the subsultus tendinum and delirium continued; and a collection of puriform mucus in the trachea and bronchi interrupted respiration, and was with difficulty expectorated, and that without cough. The mercurials were omitted; and she was ordered,

Ammon. Sesquicarb. gr. iv. ex Infusi Serpentariæ ℥i. 4tis horis sumend.

"*March* 25.—She had passed an equally restless night, with grinding of the teeth, catching at imaginary objects, subsultus, and muttering delirium: the urine was passed involuntarily. Respiration was less obstructed; but she appeared to refer to the larynx as the seat of pain: the pulse were exceedingly feeble and rapid, and the gums were decidedly under mercurial action.

"Brandy, beef-tea, &c. were administered without rallying her; and she died in the evening, but without having had any convulsions.

"*Sectio Cadaveris*, fifty-four and a half hours after death. *Head.*—The vessels of the pia-mater were much congested; numerous glandulæ Pacchioni were seen on each side of the longitudinal fissure. Beneath the arachnoid on the convexity of the hemispheres, there was some opaque albuminous effusion, with thickening of the membranes. At the base of the brain there was considerable serous effusion, as far backwards as the pons Varolii; and about the optic commissures there was much opaque albuminous deposit. The convolutions were flattened, and closely packed: the puncta vasculosa were numerous and large: the lateral ventricles contained more fluid than is usually found.

"*Thorax.*—There was congestion of both lungs posteriorly, with a circumscribed spot or two of hepatization of the size of a shilling in the right lung. There was vascular turgescence of the bronchial membrane, and a frothy mucus in the tubes.

"*Abdomen.*—The aggregate and solitary glands were prominent and vascular, but no ulceration was observed. There was considerable hepatic venous congestion.

"The remaining organs were found healthy in appearance."

Another case of arachnitis follows; but not presenting any material difference, either in symptoms or post-mortem appearances.

CASE 2. *Hydrocephalus.*—This was a delicate child, four years of age, with expanded head, and former good health and quick intellect. During the last two months complained of drowsiness—strabismus of right eye—pain in the abdomen, followed by febrile symptoms of the infantile remittent character.

"On admission, his aspect was pallid and subdued; the face looked puffy; the pupils were dilated and sluggish; he lay in a kind of quiet stupor; and complained, when asked, of pain in the forehead and occiput. The sutures were closed and ossified: the surface of the head was hot, and that of the body harsh and dry: respirations were 30 in the minute; the pulse 132, small and regular; the abdomen flat, and painful on pressure in the epigastric region; the tongue pale and white, with prominent papillæ: he had anorexia and slight thirst. The alvine evacuations were dark, scybalous, and offensive. The urine was loaded with lithates, and was slightly albuminous as tested by exposure to heat."

Head shaved—leeches to temples—calomel and castor oil, &c. He continued much the same for five days, when he became worse in all respects. Blisters—mercurial frictions. After some variations, he sunk on the eleventh day after reception in hospital.

*Dissection.*—"The convolutions of the brain were flattened and pale: the arachnoid dry; the lateral ventricles distended with serum; the foramen of Monro was enlarged and irregular; the posterior portion of the fornix softened. The cerebral substance in the other parts appeared healthy: the sub-arachnoid tissue at the base of the brain was much infiltrated, pale, and opaque.

"Tubercles were distributed through both lungs, and their edges were emphysematous.

"The right side of the heart was turgid: the mesenteric glands were hypertrophied and indurated: the other viscera were healthy."

CASE 3. *Local Paralysis—Neuralgia.*—This case was more fortunate than the preceding ones. Anne Tilson, aged 47, admitted August 7, 1843. Two days previously was attacked suddenly with pain behind the right ear, and tumefaction of the part, and numbness of the whole side of the face. The mouth was drawn to one side, and she was unable to shut the right eye. The right half of the tongue was insensible—articulation confused. Thus it appeared that the three divisions of the fifth, and the portio dura, were paralysed. Low diet—cupping on the nucha—five grains of pil. hydrarg. with fifteen grains of soda thrice a day. In two days the gums were tender. After this she improved, but, on leaving the hospital, the features were still distorted.

These affections of the facial nerves, both neuralgic and paralytic, are daily becoming more numerous.

CASE 4.—A young man had been a victim to hemicrania, after long exposure to miasmata. Every remedy was tried, including mercury, colchicum, quinine, arsenic, iodide of potassium, and large doses of stramonium, without the slightest benefit. At length, a discharge of blood and matter took place from the right ear, when the hemicrania disappeared. We observe that stramonium and arsenic were continued for some time after the abscess broke; but we confess that it appeared a useless precaution; for it was nature, and nature only, that cured the complaint.

We pass over two cases—one of scrofulous tumor in the brain, with softening, convulsions, and paralysis :—the other, a case of tubercles in the brain, as offering nothing new or interesting.

Several cases of chorea follow, but do not demand much notice in this place. Purgatives, arsenic, and sulphate of zinc appear to be the chief remedies employed at Guy's Hospital, for this troublesome complaint.

Case 5. *Hemiplegia.*—A man, aged 72, strong-built, and of florid complexion, was admitted March 20, 1843. Five years previous to admission had had scarlatina, without dropsical effusions. Two months after this, was suddenly seized with hemiplegia of the left side, not affecting the intellect, and passing off in a few days. Two days before admission, had vertigo, dimness of vision, tinnitus aurium, head-ache, &c. On the morning of admission, was seized with paralysis of left arm and leg. The mouth was drawn to the left side. The urine was coagulable, specific gravity 1008. The abstraction of a few ounces of blood caused tremor and sinking of the pulse, which rose again to 80. The motions were passed in bed. On the third day, gout appeared in the left hand. After this he gradually got lower and lower, and sloughing took place on the nates, which terminated his existence, the urine being albuminous till the last. There was no examination *post mortem.*

Case 6. *Cerebral Disease with Amaurosis.*—W. C. aged 27, married and temperate, had been subject to failure of sight at particular hours of the day, together with giddiness and head-ache. On admission the countenance had a somewhat idiotic appearance—the cardiac sounds over an increased space—pulse 72—trembling of the limbs, and sense of numbness in left arm—daily recurrence of amaurosis. This was 17th Nov. 1841. Ordered low diet and purgatives, with a seton. On the 21st there was considerable febrile re-action, requiring depletion. After this, mercury and iodide of potassium were given, and the mouth made sore. The attacks of amaurosis became less frequent, and at length disappeared. He left the hospital much improved in general health.

Case 7. *Hysterical Epilepsy.*—A girl, aged 16 years, had menstruated during the last year, once a fortnight, with leucorrhœa intervening. Three weeks before admission had a fit, apparently epileptic, preceded by sickness after eating. The fit was sudden, and she remained unconscious for more than an hour, in an outhouse, and unseen, this was followed by headache and pains in her limbs. She had a second attack, both at the approach of the catamenia. On admission, the heart's action was very irritable, pulse 100. Thoracic and abdominal viscera sound. Sulphate of zinc was administered, at first in doses of two grains thrice a day, increased to eight grains ter die, without sickness or other inconvenience. She was soon discharged cured.

Case 8. *Spinal Meningitis.*—A young man, aged 19 years, temperate in his habits, and healthy till within 18 months ago, when he had been in the hospital for pleurisy. After discharge he became affected with scarlatina, followed by wandering pains in his neck and loins, with general malaise. He had been an out-patient for the supposed rheumatic pains; but was admitted, May 7th, with symptoms of fever—pains in the neck and loins—disinclination to turn in bed—tetanic rigidity of the muscles of the back, &c. Three days afterwards he lost the use of his arms for a time, with temporary cessation of pain, but returned with motion. Next day convulsions came on, with foaming at the mouth, distorted features, clenched hands, insensibility—tonic rigidity of neck—trismus—death.

*Post-mortem.*—Skin slightly jaundiced—veins and sinuses of head large and congested—puriform fluid exuding, apparently from the centre of the spinal cord, just below the medulla oblongata—effusion of lymph and puriform albuminous matter between the arachnoid surfaces, and beneath the arachnoid itself, rendering these membranes slightly adherent and opaque. The lungs were adherent posteriorly, and congested. Liver granular and easily lacerable—spleen the same—kidneys coarse—other appearances not very particular.

CASE 9. *Tetanus.*—A young man, aged 27, a tee-totaller, had, while sifting cinders and exposed to cold, abraded the skin of two fingers. A few days afterwards symptoms of trismus, and then tetanus set in, and lasted till death, in despite of every remedy, including the Cannabis Indica. The appearances on dissection, were chiefly traces of inflammation and congestion about the brain and spinal-marrow.

We must take leave of this paper, having noticed nine cases out of 39 recorded. Many of the cases might have been left out, as not sufficiently interesting, and all of them are detailed with much more minuteness than was necessary. We have greatly abridged the more important of them for the benefit of our readers.

---

CASES OF PELVIC INFLAMMATION, WITH ABSCESS, OCCURRING AFTER DELIVERY; WITH REMARKS. By JOHN C. W. LEVER, M.D. [Guy's Hospital Reports, April 1844.]

This disease is, by no means, of infrequent occurrence, though not often treated of by systematic writers on obstetricy. Dr. Lever's paper, in a late number of the Guy's Hospital Reports, is therefore deserving of full notice. The complaint has been alluded to by HUNTER, under the name of "iliac abscess," *not* dependent on a determination of *milk* to the part—by Levret, as a "depôt laiteaux" —by Deleurye as the same—and by Kennedy as "secondary inflammation." Dr. Doherty, Dr. Churchill, and others, have also noticed the complaint in modern times.

Abroad, Dr. Martin, of Montpellier, has published an excellent monograph on the subject, and Dr. Lever has read a considerable number of the following cases before the Physical Society of Guy's, in the course of the last Winter. We shall give a condensed view of the principal of these.

*Case* 1.—Ann D——, mother of six children, was attacked with puerperal fever on third day after confinement, and treated by local depletion, aperients, &c.; but chronic inflammation remained among the pelvic viscera, for several weeks, when she came under the observation of Dr. Lever, at the hospital. She had now, bearing-down pains—weight in the pelvis—quick feeble pulse—furred tongue—restless nights—regular bowels. A distinct fulness was perceptible in the uterine region. Vaginal examination shewed heat, and induration in the left side of the vagina. A mixture of salts, senna, and hyoscyamus, with Dover's powder at night. Little alteration occurred during a week, when the abdominal swelling increased, with frequent micturition, irritable bowels, nocturnal perspirations, rigors, quick small pulse, furred tongue, depression of spirits, &c. In about a fortnight, she discharged half a pint of pus into the vagina, streaked with blood, and followed by mitigation of symptoms. The discharge continued for several days. Vagina frequently syringed with warm decoct. pap.—generous diet—pint of porter—quinine. In three weeks she was well.

*Case* 2.—Mrs. J. on the third day after delivery of her third child, had a rigor —was found with hot skin, quick pulse—lochia small—no abdominal pain or tenderness; but with sensation of weight in the pelvis, and difficult micturition. Febrifuges prescribed. The pulse continued to range high—tongue furred—milk deficient—pelvic uneasiness increased into pain, in the region of the left ovary. Vaginal examination proved this canal to be hot, hard, inelastic. Local anodyne injections—hyd. cum creta with opium—antifebrifics—leeches thrice repeated. In three days a copious purulent discharge from vagina, which lasted ten days. A pint of porter daily, with generous diet, and bark. Phlegmasia dolens succeeded, treated by leeches, fomentations, &c. The phlegmasia afterwards attacked the other side, with great violence. Convalescence was slow.

*Case* 3.—On the morning of the thirteenth day after delivery, was attacked with rigor, followed by considerable re-action—pain in the left iliac region—pulse 120, small—furred tongue—pain on pressure in left side of abdomen. Twelve leeches to the painful part—hot anodyne fomentations—calomel, castor-oil, antifebrifics. Amelioration of symptoms; but subsequent exasperation, and recourse to antiphlogistics, with mercury till the gums were affected. In the course of a few days purulent fluctuation just above Poupart's ligament, left side. On incision, six ounces of matter were discharged, very fœtid. The discharge continued eleven days. She took quinine, porter, and the mineral acids. Her convalescence was protracted for two months, when she recovered.

In the *fourth* case, the abscess also burst externally on the right side, the abscess being preceded by the usual symptoms of suppuration. The patient refused to have the tumor opened; but it burst and discharged for a month, when she recovered.

The *fifth* case was distinguished by a double abscess, one of which burst into the vagina, the other was punctured, and the patient recovered.

The *sixth* case was a primipara, where the abscess burst into the vagina, five weeks after delivery. Recovery took place.

Nine cases are related altogether; but the above are sufficient samples of all. The following judicious remarks, by the author, are worthy of record here.

1. "*Seat and origin of the disease.*—It is without doubt a very difficult question to decide whether the disease in the foregoing cases commenced in the uterine appendages, strictly so called, viz. the ovaries, Fallopian tubes, and broad ligaments, or whether the cellular tissue was the structure primarily affected. In the only case I had the opportunity of examining after death, the evidences of previous mischief were found in all these structures (see Case 8): and it is hard to conceive that one can be seriously implicated without the participation of the others. That the cellular tissue of the pelvis is involved, will, I think, be readily granted, after a careful perusal of the cases. The tumefaction felt both externally and internally; the hard, tense, inelastic condition of the vagina; and the channels by which the pus evacuates itself, its effecting its exit *without* the peritoneum; prove to my mind, very satisfactorily, that in such cases the tissue which intervenes between the pelvic fascia and serous membrane is involved.

2. "*Causes.*—This disease may follow an attack of acute inflammation; or it may remain as the sequent of puerperal fever (see Case 1). In the greater number of the previous cases, anomalous symptoms displayed themselves soon after delivery. Cold, falls, blows, &c. are said to have produced the disease. In but two cases were the labours unnatural; viz. Case 3, in which the operation of version was performed; and Case 5, delivered by the forceps. In two cases the right side of the pelvis was affected; in five the left; and in two matter was evacuated from both sides: in one, the right preceding the left; in the other,

Case 9, the pus was evacuated from the left side externally, and from the right side, through the bowel.

3. " *Symptoms : General.*—The symptoms may commence a day or two after delivery, or they may supervene some days and even weeks after labour. The disease is mostly preceded by rigors, or a sensation of coldness over the surface; followed by heat of skin, quickened circulation, and pain in the region of the pelvis. The febrile paroxysms may remit, but their intervals of recurrence are at varying intervals. The uneasiness and pelvic pain continue, and, as the disease progresses, increase: usually there is some degree of stiffness in the side affected, and not unfrequently pain in the course of the vessels of the thigh and leg: this may proceed to the development of phlegmasia dolens (see Cases 2, 6, and 7). The pulse is seldom below 100—110: the tongue remains loaded; there are frequent calls to pass the urine, which is scanty and high-coloured: at one time there is constipation of the bowels, at another, diarrhœa associated with tenesmus; and the secretion of milk is usually scant, or altogether suppressed.

" These symptoms may continue for an indefinite period; when, if the disease be overlooked, or if the remedies employed do not succeed in checking its progress, they are followed by the attendant signs of suppuration, and the matter may be evacuated either by an artificial or natural opening."

The patient generally directs to the seat of the disease, where will be visible or tangible a swelling, or a hardness, very tender to the touch, and very variable in extent. On examination, per vaginam, we shall sometimes find no indication of disease—but often " *a morbid permanence of the state of puerperal hypertrophy ;* " to use the quaint and somewhat mystified expression of Dr. Simpson. " As a general rule, it will be found, that wherever pelvic inflammation occurs soon after delivery, a long period will elapse before the uterus returns to its original state. In other females, the upper part and side of the vagina will be found hard, tender, firm, and inelastic; and, by pressing upon the swelling felt through the abdominal parietes with one hand, and keeping the forefinger of the other in the canal, we are able to satisfy ourselves that the hardness and swelling felt in both situations arise from one and the same cause."

*Diagnosis.*—The disease may be confounded with abscess seated in the parietes of the abdomen, caused by rupture of some muscular fibres during parturition, or without any mechanical cause. " It will be well, therefore, to mark the diagnosis between simple abscess of the abdominal walls, and those collections of matter which issue from the pelvis behind, and external to the peritoneum, presenting themselves in either iliac region. In the early stage of the latter, the skin, as well as the muscular parietes, may be readily rolled over the tumor; evidently demonstrating their non-connexion: while, if the abscess be seated in the abdominal walls, by moving the one we move the other. This method of diagnosis is most satisfactorily applied when the patient is in a prone position."

Morbid enlargements of the uterus, continuing after delivery, may be confounded with the malady in question. In these cases, Dr. Lever thinks that much valuable assistance may be obtained from explorations by Professor Simpson's Uterine Bougies.

*Feculent Collections.*—It is not so very easy to distinguish accumulations in the caput coli and in the sigmoid flexure, from ileo-cæcal and other pelvic abscesses, since, in fact, those said accumulations too often produce inflammation, suppuration, and ulceration. Our author, however, thinks otherwise.

" The early period at which they occur after delivery; the tympanitic condition of the abdomen; the frequent expulsions of flatus, both by mouth and anus; the frequent colicky pains; the occasional vomiting; the loaded tongue; the state of the pulse; will enable us to frame a correct diagnosis. And further, upon inquiry, we shall find that for some time the patient's bowels have been in a constipated state; while the exhibition of purgatives, and the administration of cathartic glysters, by their effects will remove all doubt from the case."

We shall not dwell on the diagnosis between the disease in question, and sciatica and some other affections with which it has been confounded. The history and symptoms will generally lead the practitioner to a proper conclusion.

*The Termination* is sometimes by resolution, when the disease is early treated; but more frequently it proceeds to suppuration, pointing outwardly—into the cavity of the peritoneum—into the vagina—or into the uterus—and into the bladder or intestines.

*The Sequelæ* are immobility of the uterus—an impervious condition of the Fallopian tube or tubes—ovarian disease, &c.

*Treatment.*—The great object is resolution; but as the patients are not generally in a state to bear heroic remedies, the milder means will be preferable. Leechings will be generally preferable to venesection. They may be applied either to the seat of pain, or to the vagina, with warm fomentations or cataplasms. Mild mercurial preparations, in small doses, just to gently affect the system, will be beneficial. The kidneys and the bowels must be kept in action—and diaphoresis promoted. The suffering of the patient must often be attended to and relieved by opiates, either by the mouth, or as suppositories.

If suppuration cannot be avoided, it must be promoted, and then the constitution supported.

" 1. The first object will be accelerated by the continued application of medicated poultices and fomentations, which soothe the pain, and lessen the patient's sufferings. When the symptoms plainly indicate the formation of matter, and fluctuation is evident, the abscess should be opened: this may be done externally, through the abdominal parietes; internally, through the vagina, by means of the speculum and a guarded lancet; or through the rectum, by means of a trocar, as in Dr. Simpson's case.

" M. Martin recommends the application of caustic potass to the abdominal parietes, for the double purpose of having an external opening and securing previous adhesion of the containing sac to the abdominal walls. This method of practice appears to have been very successful in his hands; but still there are cases to which its inapplicability must be obvious.

" In some of the cases related, the patient's sufferings would have been diminished had an earlier opening been made.

" 2. The constitutional powers must be maintained by a generous, nutritious diet, porter, wine, &c.; the administration of tonics, and sedatives.

" Where inflammation takes place in the course of the veins and absorbents, leeching along the line of the inflammed vessels, followed by hot spirit fomentations, will be found of service; taking care, at the same time, to allay pain and irritation by sedatives, and to support the system by mild tonics.

" In some cases, where the inflammation does not proceed so far as suppuration; and in others, where pus has formed and has been evacuated; there will remain considerable induration of the affected structures. Its absorption will be promoted by the exhibition of the pot. iodid. two or three times a day, in the dec. sarzæ c., or the dec. cinchonæ, and by the application of blisters."

This paper of Dr. Lever, is equally creditable to himself, as a practical physician, and to the character of the valuable publication in which it appears.

---

### On Paracentesis Thoracis. By Messrs. Dr. Hughes, and Edward Cock. [Guy's Hospital Reports.]

In the April Report of the work above cited, is an interesting memoir on the operation in question, and on its comparative success. The paper opens with general observations on thoracic effusions requiring the operation. The authors have found the operation more easy, and much more efficacious than has been represented in writings. Dr. Bennet states that Boyer had never been successful in Paracentesis Thoracis, while Dupuytren had seen only two favourable cases out of fifty. Sir A. Cooper met with only one fortunate case. We need not go farther for unsuccessful terminations in paracentesis thoracis; and it is our pleasant duty to turn to a brighter page in the history of the operation.

Within the last four or five years, paracentesis thoracis has been performed at least from twenty to thirty times in Guy's Hospital. The operation was resorted to, merely for temporary relief; and though life was not ultimately saved, in all cases, great mitigation of suffering was obtained. The diseases for which paracentesis may be resorted to, are hydro-pericardium—sanguineous effusion into the pleura—hydrothorax—pneumo-thorax—and empyema.

Our authors are of opinion that the operation in pneumo-thorax can hardly ever be successful, but only to be employed to relieve urgent dyspnœa. The same observation, however, applies to the operation in all cases. Hydrothorax is rarely or never an idiopathic affection, but the effect of some disease—too often an organic one, and therefore beyond the reach of medicine. But the dreadful sufferings from dyspnœa are almost intolerable, and the relief from them by tapping is well worth the risk and pain of the operation.

"The indications, then, for paracentesis in empyema, or chronic pleuritic effusion, appear to be, in the first instance, the presence of a large quantity of fluid in the pleura rapidly effused; in the second, the distress of the patient dependent on the great accumulation of fluid; and, in the third, the existence of a considerable amount of effusion, together with such a state of constitution, or of the general health, or such other circumstances, as would render a prolonged purely medical treatment injurious or undesirable."

We shall here abbreviate some of the cases adduced by our authors, but first introduce a passage from Mr. Cock, respecting the operation itself.

"It now only remains for me to describe the operation itself; which, as regards the pain it inflicts, is so trifling, that by avoiding all unnecessary display and preparation, the patient may be led to consider it as little more than the sequel of the discipline to which he is occasionally subjected when it is considered essential to make a thorough examination of his chest; the same position of the body being alike adapted for the one process as for the other. It will be found most convenient to let the patient sit *across* the bed, so as to admit of his body being readily lowered and supported over its edge. The spot having been determined upon, it is advisable to make a small puncture in the skin, just at the upper edge of the rib, with a narrow-bladed lancet; through which opening the exploring needle and subsequently the trochar may be inserted. This preliminary step is not absolutely necessary; but as the skin is by far the most impenetrable and resisting of the tissues to be traversed, its previous division will render the introduction and withdrawal of the canula more easy, less forcible, and attended with a minor degree of pain and alarm to the patient. The exploring needle having been first introduced and the presence of fluid ascertained, the trochar and canula may then be carried into the chest through the same track, giving the instrument a slight obliquity upwards, which will enable it to clear the edge of the rib. The depth to which the trochar must be passed will of course depend much on the thickness of the parietes, the presence of fat, muscle, or œdema, for which due allowance should be made; and, in most instances, the penetration of the pleura will be appreciated by the sensation conveyed to the fingers of the operator, especially if the integument has been previously incised so as to diminish materially the friction.

"The remainder of the operation consists of getting rid of as much fluid as the strength and condition of the patient will bear, and carefully avoiding the admission of air into the cavity. On withdrawing the trochar, the fluid will at first be found to flow in a steady and equable stream, slightly augmented in force at each expiration. After the lapse of a shorter or longer period, the flow will become checked at each inspiration, and then the body of the patient should be gently lowered into an horizontal posture, and turned slightly on to the affected side, so as to bring the cavity directly over the opening; and in this position he should be duly supported by assistants. The fluid will now recommence flowing in an uninterrupted stream; and when it again begins to flag, a still further quantity may be obtained, if the state of the patient permit it, by directing an assistant to make steady and continuous pressure on the lower part of the chest, by grasping it on either side with the hand."

During the process of evacuation, great attention should be directed to the stream of fluid, which should never be allowed to be completely interrupted during inspiration—the admission of air being immediately indicated by a sucking noise, which cannot be mistaken, and which is the signal for a prompt withdrawal of the canula.

*Case* 1.—J. P. aged 22, admitted (July 27, 1843) under Dr. Addison, having been subjected to cold while taking mercury for venereal complaints. Ten weeks ago, attacked with pain and tension in the epigastrium—eructations—tenderness on pressure, &c.—succeeded by short and oppressed respiration, hard cough, and mucous expectoration, which continued till admission, when the nose, lips, ears, and fingers were blue or rather livid, with dyspnœa—sense of abdominal fulness, and difficulty of lying on the right side or back. Tongue clean, appetite good, skin dry, bowels regular, evacuations bilious, urine natural, pulse 100, irregular, small, and feeble. Left side of chest more rounded and full than right side, little raised on inspiration, and tender on pressure in the intercostal spaces. It was universally dull on percussion—absence of vibration on speaking. The integuments were œdematous. The respiration was nul on the inferior portion of that side—became tubular as the stethoscope was passed upwards, and puerile, with sonorous sibilant rhonchi above the spine of the scapula. Below the scapula the voice was bronchophonic—and over that bone, egophonic. Nothing remarkable on the right side. The impulse of the heart could not be felt to the left of the sternum, and its sounds were most distinctly heard over the centre of the right side of that bone. Fluctuation in the abdomen—loins and legs œdematous—liver felt below the ribs. To be cupped, and take calomel, opium, and antimony. In four days the gums were affected, and no relief being obtained, fifty ounces of clear straw-coloured fluid were drawn off by the trochar. On cooling it presented a large loose transparent coagulum, floating in some clear serum. After this he had copious expectoration, sometimes bloody, sometimes muco-purulent, with various alterations, for better or worse. Mercury, diuretics, salines and other medicines produced little effect. In consequence of great dyspnœa and distress, the chest was tapped thrice, and on each occasion produced great relief. Afterwards ascites took place, for which he has been several times tapped. On the 6th March, 1844, nearly nine months after coming under treatment, he is able to walk about the ward—can lie, and occasionally sleeps on the right side; but still the effusions continue, the enlarged liver is felt, and there can be little or no hope of final recovery. There can be no doubt, however, that, had it not been for the operation, he would have died long ago.

*Case* 2.—W. C. a coachman, aged 27 years, had been in Hospital for secondary venereal eruptions. On the 1st Dec. 1842, Dr. Hughes was called to him, in consequence of dyspnœa, without cough or fever—tongue clean, skin natural, pulse feeble. The heart could not be felt in the usual position—its impulse felt on the *right* side of sternum.

"The entire left side, both before and behind, *i. e.* as high as the clavicle anteriorly and the spine of the scapula posteriorly, was quite dull on percussion. No respiration could be heard at any part of this side, except immediately below the clavicle before, and in the interscapular region behind, where it possessed a pure tubular character. The voice was more shrill than natural in the left interscapular region, but was indistinct below the clavicle, and scarcely at all audible in any other part of the side. Mr. Cock, at my request, performed the operation of paracentesis; and, having first used the explorator to certify the presence of fluid, introduced a small trochar at the posterior and inferior part of the left side of the chest. About six ounces of clear greenish-yellow serum were drawn off, with slight relief to the patient. Ordered,

Pil. Hydrarg. gr. iv. Opii gr. ss. m. ft. Pil. nocte maneque sum.

Liq. Potassæ m. xv. Potassii Iodidi gr. iij. Inf. Gentian. C. ℥iss. m. ft. haustus ter die sumend.

"*Dec.* 2—This day his pulse were considerably fuller and stronger, and there existed some heat of skin, with a flushed face. In other respects he was the same. Ordered,

Ven. Sect. ad ℥viij.—Empl. Lyttæ lateri sinistro.—Pergat.

"*Jan.* 6.—The febrile symptoms were immediately and permanently subdued by the venesection; but his dyspnœa and other symptoms continued nearly as before, and the physical signs of his complaint were unchanged. He had hitherto continued the medicines first prescribed: the blister had risen and discharged freely. He was certainly not better, though not considered materially worse; but late in the evening of this day Mr. Cock and I were called to him, in consequence of excessive dyspnœa threatening suffocation, arising from the increased accumulation of the effused fluid. Upon our arrival he was gasping for breath, propped up in bed by pillows, with much anxiety of expression, a clammy state of the surface and feeble pulse. The chest was again tapped at the same part as before; and, by the assistance of pressure upon the ribs, thirteen ounces of the same kind of fluid as before were evacuated. In a few minutes he felt no inconvenience whatever from the operation, and in a very short time expressed himself as being much relieved thereby. From this time his recovery commenced."

The blister and other remedies were continued, the dulness gradually decreasing and breathing easier. The respiratory murmur is gradually returning to all parts of the lungs—mouth tender from mercury. From this time he continued free from the thoracic symptoms, though he was twice obliged to return to the hospital for venereal eruptions. This is decidedly an excellent case. The recovery seems to be complete.

*Case* 3.—E. M. an Irish sailor, aged 58, who had been at the battle of Algiers, admitted 7th April 1842, for fractured ribs. He had been run over by a cart. In a fortnight he was made an out-patient; but a day or two afterwards, was seized with great oppression of breathing, anxiety of countenance, sense of suffocation, &c. On removing the bandage, the left side was unnaturally resonant on percussion. The respiratory murmur deficient in the left side—nothing remarkable in right side. Venesection to six or eight ounces—calomel, opium, and antimony. It was considered that pneumo-thorax had taken place in the left side, from laceration of the lung. To be bled to eight ounces—opium to be given every six hours, with mild nourishment. He improved progressively, and in a few weeks, was able to walk about. After this, he again complained of pain in the right side, where there was dulness, and want of costal elevation. Pleuritic effusion was suspected. Blue pill and opium at night—a cathartic in the morning—and iodine in the day. He got worse—muco-purulent, and ultimately purulent expectoration took place, and it became evident that fluid was accumulated in the right side. The explorator was introduced, but no fluid came forth. He lingered for nearly three months, and then sank.

*Dissection.*—The instrument was found to have neither penetrated the lung, nor to have entered any collection of fluid. It had entered the diaphragm. There were extensive pleural adhesions. Both lungs appeared sound *in situ;* but on taking off the coats of adhesive matter, the finger passed into a narrow and defined cavity in the right pleura, containing three or four pints of sero-purulent fluid. It contained no air. The cavity was lined with a dense layer of coagulable lymph. The lung itself was firm from compression, and a part was solid. There was a communication found between the extremity of a bronchial tube and the defined cavity in the serous membrane above alluded to. There was a small cavity capable of containing a pullet's egg in the apex of the *left* lung, filled with a dark-coloured fluid.

"Whether I was correct in the opinion I expressed as to the presence of pneumo-thorax upon the left side in this case, and the air was subsequently absorbed, must remain undetermined. The existence of a cavity close to the surface of the lung renders it not improbable that such had really been the case, particularly when taken in connection with the sudden supervention of symptoms which usually accompany that accident. Whether the patient would have been permanently benefited by the evacuation of the fluid effused on the right side must also continue uncertain; though the very little disease existing in other parts induces me to think that such a result might have followed a more successful operation." 87.

*Case* 4.—A girl, aged nine years, was brought to the hospital as an out-patient, (Sept. 7, 1843,) but was admitted under Dr. Hughes. Five weeks previously was attacked with inflammation of the bowels. A fortnight before admission was seized with severe pain in the left side, catching her breath—also pains in the head and limbs. She had cough and large expectoration, bowels open. On examination, the respiration was much hurried—dyspnœa—pulse 140.

"*Physical Signs.*—Enlargement and fulness of the left side were obvious upon inspection. The intercostal spaces were level with the ribs, which were but slightly elevated during inspiration, and were thereby forcibly contrasted with those of the opposite side. The circumference of the chest measured twenty-three and a half inches; and by passing a tape below the nipple, from the spinous processes to the centre of the sternum, the admeasurement of the right side was found to be eleven inches, while that of the left reached twelve inches and one-third. There existed general dulness of the left side on percussion, together with absence, or great distance of respiration, and of the voice, though these could be but imperfectly investigated from the continual restlessness of the little patient. The heart could not be felt, though it was indistinctly heard below the left nipple; it was both heard and felt on the right of the sternum. The dulness on percussion existed up to, and indeed was most marked in its character below, the left clavicle, in which situation tubular breathing and imperfect pectoriloquism could be distinguished. The right side presented nothing remarkable, excepting the position of the heart."

Next day Mr. Cock introduced the explorator in the left side. A few drops of pus escaped, while some purulent expectoration appeared from the mouth. On the introduction of a small trochar a jet of serum escaped—first limpid and then tinged with blood. The canula was withdrawn. From this time she gradually, but entirely recovered.

The author candidly states that he doubts whether the operation, in this case, did any good. It is quite evident that it did no harm. It also shewed that the operation is not necessarily a dangerous one.

*Case* 5.—This was one of partial recovery from hydrothorax, ascites, diarrhœa, phthisis. Paracentesis twice performed. The patient was a female, aged 23, and liable, from childhood, to cough. She was the mother of four children.

About eleven weeks before admission, the abdomen began to enlarge, and the cough was more troublesome. She could only lie in the semi-erect position—urine scanty and high-coloured—great dyspnœa—pale face—tongue clean—emaciation—diarrhœa—abdominal fluctuation—no tumor of liver or spleen—pulse 140, feeble.

"The entire chest moved imperfectly upon inspiration. Marked dulness existed anteriorly upon the right side as high as the mamma, and posteriorly upon the left side up to the angle of the scapula. Absence or distance of the respiration, and decrease of the normal resonance of the voice, were obvious in the latter situation. Below the clavicles, but especially below the right, the respiration was hoarse, and mixed with mucous rattles. The impulse of the heart was felt nearer to the sternum than is usual; but the rhythm of the organ was normal, and the sounds were scarcely otherwise. As the diarrhœa required immediate attention in her weakened state she was ordered,

Pulv. Kino C. gr. x. omni nocte sumend.; et Pulv. Cretæ C. c̄ Opio gr. x. ex. Mist. Mucilag. ter die; with Arrow-root and Beef-tea for diet.

She continued without much alteration till the 28th; when the diarrhœa having been checked, though the tongue was still red and scabrous, the urine in very small quantity, the dyspnœa increasing, and decubitus equally or more difficult, she was ordered,

Potassii Iodidi gr. ij. Liq. Potass. m. xv. Inf. Gentian. C. ℥iss. ter die.—Rept. Pulv.

"*Oct.* 29. The dyspnœa and orthopnœa having increased rather than diminished, and dulness on percussion, together with absence of the respiration and the voice, nearly perfect immobility and slight enlargement of the left side, and partial displacement of the heart having been distinctly ascertained to exist, Mr. Cock, at my request, introduced, first the explorator, and then a small trochar, between the eighth and ninth ribs posteriorly, and drew off seven ounces of clear, yellowish serum. The patient felt almost immediately relieved. On the next night she was able to lie much lower in bed. The left side was found less extensively dull on percussion, and the heart less displaced towards the right side. Pleuritic rubbing was now distinctly heard below the left clavicle; the cough was still troublesome; the expectoration scanty and mucous; but the urine was considerably increased in quantity. Pulse still 140."

The dyspnœa having again increased, the trochar was introduced, and fifteen ounces of slightly sanguineous serum were drawn off, with great relief. In the course of a fortnight she was so far recovered, as to leave the hospital and return to her friends. "She was able to lie down in bed, and to turn upon either side—her appetite was good—improved in strength and flesh—pulse still 128." Dr. Hughes saw her a fortnight after she left the hospital, and she continued much in the same state.

Although this cannot, of course, be pronounced positively as a case of recovery; yet it is not very far from it, and is well worthy of record. We can only make room for one more case.

*Case* 6.—H. S., aged 19 years, had been exposed to cold, in wine-vaults, and experienced an attack of pleurisy two years previously. Admitted Dec. 2, 1840, with cough and expectoration. He is emaciated—nocturnal perspirations—dyspnœa—œdema of the ancles—entire right side dull on percussion—the left resonant, with puerile respiration. Can only lie on right side. In the night of the 15th December he was suddenly seized with violent cough, which continued nearly half an hour, with great sense of suffocation. The expectoration was bloody. Dr. Babington believing that the pleura contained fluid, Mr. Cock introduced the explorator, and afterwards the trochar, when two pints of purulent fluid were drawn off. This was followed by another violent fit of coughing, by which his life was endangered. For some weeks he daily expectorated nearly a pint of matter, apparently containing shreds of hydatids. In about two months he began to cough less, and to gain flesh. He shortly afterwards left the hospital in tolerable health, and was seen more than a year after dismissal, perfectly well.

We have thus given our readers a full abstract of six out of ten cases recorded in the work before us; and we can hardly believe that they will differ from us in the opinion, that the said paper is worthy of the authors, and of the valuable work in which it appears.

---

Observations on Lithotomy.—[Guy's Hospital Reports, April, 1844.]

In the first volume of the Reports, Mr. Cooper communicated some observations, of a valuable practical character, on Lithotomy, which we noticed at the time. In the present number, we find a continuation of the paper.

Ten cases are first related, the nature of which may be gathered from their heads:—

Case I. *Calculus Vesicæ—Operation. Urine passed through natural passage on third day—Rapid Recovery.*

Case II. *Calculus Vesicæ—Symptoms excessive, but relieved by Medicine—Operation—Recovery.*

Case III. *Calculus Vesicæ—Operation—quite well in three weeks.*

Case IV. *Calculus Vesicæ—Operation—Recovery.*

Case V. *Calculus Vesicæ—Small Stone—Water passed by Natural Passage six days after Operation, and Patient quite well at the end of three weeks.*

Case VI. *Lithotomy after Lithotrity.*

Case VII. *Calculus Vesicæ—Strumous Constitution—Small Stone—Water passed by Natural Passage eight days after the Operation.*

Case VIII. *Calculus Vesicæ—Operation—Large Stone—Recovery.*

Case IX. *Calculus Vesicæ—Lithotomy after Lithotrity.*

Case X. *Urinary Calculus—Sequelæ of Typhus Fever—Operation—Recovery.*

We don't see that it is necessary to narrate cases whose titles speak so intelligibly for them. Amongst other observations of Mr. Cooper's we find the following:—

"As regards the patients best adapted for the operation of lithotomy, little doubt can be entertained but that its success is much greater in children than in those who have arrived at the period of puberty; and that persons from the age of twenty-four to forty-five bear the operation worse than at any other period of life. The favourable result of the operation in children has been attributed to the smaller size of the vessels of the perinæum, and of those of the organs of generation. I think, however, this opinion is derived more from reasoning than from observation; as in those who die after the operation of lithotomy, a great majority, hereafter to be shewn, will be found to have died from peritonitis, connected with some diseased state of the viscera, and chiefly of the kidneys. And, again, during the middle period of life the whole arterial system is in full vigour; and a want of balance in any of the vital functions will suffice for the development of inflammation.

"Little need be said as to the after-treatment, beyond what has already been considered; as nothing more requires to be done than paying attention to the secretions, the natural performance of which may be considered as constituting a healthy action: and perhaps there is more danger in interference during Nature's process of reparation after surgical operation, than there is in leaving them wholly to their own constitutional powers."

It appears that Mr. Cooper has operated eighty-four times at Guy's Hospital, and upwards of twenty in private; and that he has lost ten patients—a good share of success.

After the preceding cases, Mr. Cooper relates seventeen others, of a more complicated kind. He observes very justly that :—

" It must appear evident to every one, that the practice of one surgeon, however extensive his opportunity may be, will not suffice to form a complete list of the various difficulties and complications in the operation and after-treatment of stone cases; and it therefore behoves every one who has had experience in this branch of his profession candidly and boldly to state the various circumstances which have occurred to him as impediments in the operation, or led to an unfavourable result. It is not sufficient that fortunate cases only should be published, without reference to unsuccessful results; as otherwise the inexperienced may be induced to believe that lithotomy is an extremely easy operation; and if in their own practice, under such delusion, any thing untoward should occur, they will be by far less capable of combating the difficulties, than if they had been made acquainted with the obstacles which they are to expect as liabilities in the operation. When we consider the great difference in the ages and constitutions of persons operated on for the stone, the variety of calculi both as to composition and size, and the variations in depth and condition of the parts cut through, it cannot be supposed that an accurate opinion can be given from *à priori* reasoning. In the operation of lithotomy, the surgeon should bear in mind that he must be, however cautious, more or less in the dark as to the difficulties which may present themselves; and it is only by meeting them with calmness and deliberation that the operator can ever hope to encounter them successfully."

*Case* 11, the first of this second batch, was that of a boy three years of age. A short pair of forceps was introduced, but the stone was found to lay a considerable way back, and on attempting to open them, it was found impracticable; they were withdrawn, and re-introduced, but with the same result. A longer pair of forceps were then used, and the stone removed. The child recovered without any unfavourable symptom. Mr. Cooper observes upon the case :—" Those I employed at first were the usual ones intended for children; but I have so frequently found them of an inefficient length, particularly in the instance of a capacious bladder, that I am in the habit of using larger forceps than are generally recommended; and have found considerable facility both in seizing and handling the stone, from the greater length of these instruments."

*Case* 12. A boy, aged 4½ years. The stone was supposed to be large—the urethra was small, and the staff necessarily so. It was remarked that the first incision was large; but yet, from the depth of the perinæum, and the smallness of the groove in the staff, there was some difficulty in introducing the point of the knife; which, on being effected, was smoothly and readily passed into the bladder. Mr. Cooper then introduced the fore-finger of the left hand into the bladder; and finding that the prostate grasped his finger, was satisfied that the opening was not sufficient for the size of the stone; he therefore considered it prudent to enlarge the internal incision; when the forceps were introduced, and a rough stone, about the size of a walnut, was immediately removed, without any difficulty. It was observed that not a drop of urine followed the introduction either of the knife or of the forceps. The boy got quite well rapidly.

Mr. Cooper considers, and probably with reason, the making the incision in the prostate too small a fault on the right side. He remarks :—" Many surgeons, I believe, are of opinion that less injury is likely to occur from an incised wound, even if it be a little too large, than from a lacerated one, occasioned by drawing the stone through the resisting prostate. But on this point I feel perfectly assured, that the incision through the prostate, made by the knife, should be no larger than just to admit the forceps; and that the finger of the operator, and the opening of the blades of the forceps, will be found the safest, and therefore fittest means of sufficiently enlarging the aperture for the extraction of the stone. If the knife be used for this purpose, there is great danger of the pelvic fascia being cut; and the ill effects resulting have already been dwelt on, as one of the most frequent causes of the failure of the operation." We should conceive that, at this time of day, there cannot be a second opinion upon this point.

*Case* 13, that of a boy between five and six years of age. The calculus was brittle and broke. The remaining fragments were afterwards removed with a more appropriate pair of forceps, and likewise by means of a scoop. The bladder was then injected twice; and a good deal of fetid gravelly matter escaped with the water. We quite agree with Mr. Cooper, that the necessity for the frequent introduction of instruments into the bladder, under these circumstances, demands the greatest gentleness in their manipulation.

*Case* 14 was one of calculus impacted in the urethra, behind the scrotum. It was cut down on, and removed with success.

*Case* 15, that of boy aged 10. The urine was albuminous—the stone was about the size of a pigeon's egg. He recovered slowly but perfectly. Mr. C. remarks that "the urine may present the appearance of an albuminous deposit, by the application of heat only, from its alkaline condition, and without any disease of the kidneys; or it may present a similar appearance when nitric acid is applied, from the presence of lithic; and in every case, therefore, it is necessary to employ both heat and acid as tests of the state of the secretion."

In the sixteenth case, there was slight hæmorrhage.

*Case* 17, that of a boy aged 5½. The stone was small—more hæmorrhage than usual at the time of operation, but it ceased on his being put to bed; it recurred, however, about a quarter of an hour after, and apparently proceeded from the artery of the bulb; there was considerable difficulty in stopping it, owing to the extreme irritability of the child, who was constantly crying for two hours subsequent to the operation. A piece of sponge was placed in the wound, and pressure was made, and kept up, by the finger of the dresser. Syr. papav. ʒj. was given him; and he was ordered to be kept quite quiet. About two hours after, the dresser having left him, the sponge was forced out by the cries of the child. The countenance was now blanched, with dark areolæ round the eyes; the pulse was hæmorrhagic; and the feet cold. The sponge was replaced, the pressure again applied, and strict orders were given that he should be closely watched: warm flannels were applied to the feet, and another dose of syr. papav. was given him. On the 27th and 29th, hæmorrhage recurred to such an extent as almost to destroy the patient; but was however again restrained by pressure. From this period, by the administration of tonics and generous diet, he rapidly improved in health, and left the hospital on the 6th of December quite well.

*Case* 18, that of a man aged 25. Operation on the 21st June, 1842, when a large deep arterial branch was divided, and bled rather freely. Free hæmorrhage followed the operation. Constant pressure on the ramus of the left ischium was made, by means of a piece of sponge, and the introduction of the fore-finger into the wound. This was continued for about three quarters of an hour: lint, dipped in cold water, was then applied, and pressure made with a cork.

On the 25th, whilst straining to pass his urine, hæmorrhage again commenced; but was quickly stopped by the introduction of a sponge-plug into the wound, and placing a bottle of cold water between the thighs. He was dismissed cured on the 3d of August.

"These cases require all the care which an attentive assistant can bestow; as, in many instances, the loss of a small quantity of blood may turn the scale; and unless a person be at hand to apply his remedies immediately, a life, which might otherwise have been saved, is lost. The keeping the patient cool, by free ventilation of the room, the administration of cold drinks, the application of ice to the perinæum, and pressure upon the bleeding vessels, are the principal means indicated.

"I consider, however, the danger of bleeding may be much diminished by the mode of operating followed by the surgeon; and that if the urethra be not laid open in the bulb, there is but little fear of any important loss of blood: while, on the contrary, if the groove of the staff be cut down upon, anterior to the deep fascia of the perinæum, the artery of the bulb must inevitably be wounded. The operation is doubtless rendered more difficult by this deep cut into the urethra, but the safety of the step is a sufficient compensation for its adoption."

*Case* 19. A man, aged 27, had had symptoms of stone for twenty years. After the first incision, considerable hæmorrhage took place, owing to the large size of the superficial vessels: some difficulty was experienced in seizing the stone, from its large size and form. After the operation, which occupied about two minutes, hæmorrhage, to the amount of two or three ounces, occurred; which was checked by making pressure upon the perinæum with ice in a bladder. He complained of being faint, and felt inclined to vomit: pulse 82, feeble. Pressure was afterwards removed, but the ice continued to be applied.

Hæmorrhage recurred on the 28th and 30th, and August 1st and 2d, without any apparent cause, to such an extent, as considerably to lessen the hopes of recovery; but, by applying ice to the perinæum, and the introduction of a plug into the wound, it was completely stopped; and he eventually quitted the hospital perfectly well.

The calculus was of the mulberry kind.

*Case* 20. A shoemaker, aged 40, had had stricture five years previously, and laboured under incontinence of urine. Ten days before admission, tumefaction and redness were observed at the umbilicus; to which he applied some shoemaker's wax: about ten or twelve hours after its application, about a pint and a half of urine passed off, in a full stream, from a small orifice in the umbilicus, now for the first time observed; and the flow through the urethra then stopped. At the time of admission into the hospital, on the 8th day of January 1830, his symptoms were, an alternate flow of urine from the orifice of the umbilicus and urethra; scanty in quantity from the latter, very turbid, and of a reddish colour, apparently from an admixture of blood: pain, on pressure, over the right lumbar region; and, moreover, on pressing the right iliac region, an irresistible desire to micturate was induced. His general health good: cutaneous perspiration scanty. The catheter has been daily introduced, and small quantities of urine been drawn off.

Pressure over the umbilicus failed to arrest the discharge through it. A metallic elastic catheter was directed to be worn continually in the urethra. This had a great effect on the umbilical fistula. In July, however, the instrument broke, leaving about three inches in the bladder. Extraction with the urethral forceps was attempted in vain. On the 3rd August, Mr. Cooper performed an operation for its removal, which was in every respect similar to the lateral operation of lithotomy. The second incision opened the membranous portion of the urethra, and divided a part of the left lobe of the prostate gland: several attempts were now made to seize the portion of catheter, by means of the urethral forceps, but ineffectually. The prostate gland was then completely divided, and the bladder opened; and after a few efforts, the broken portion of catheter, about three inches in length, was extracted with the lithotomy forceps.

The patient recovered.

